# Novel Investigations of Flavonoids as Chemopreventive Agents for Hepatocellular Carcinoma

**DOI:** 10.1155/2015/840542

**Published:** 2015-12-16

**Authors:** Chen-Yi Liao, Ching-Chang Lee, Chi-chang Tsai, Chao-Wen Hsueh, Chih-Chiang Wang, I-Hung Chen, Ming-Kai Tsai, Mei-Yu Liu, An-Tie Hsieh, Kuan-Jen Su, Hau-Ming Wu, Shih-Chung Huang, Yi-Chen Wang, Chien-Yao Wang, Shu-Fang Huang, Yen-Cheng Yeh, Ren-Jy Ben, Shang-Tao Chien, Chin-Wen Hsu, Wu-Hsien Kuo

**Affiliations:** ^1^Department of Internal Medicine, Kaohsiung Armed Forces General Hospital, Kaohsiung 80284, Taiwan; ^2^Department of Internal Medicine, Tri-Service General Hospital, National Defense Medical Center, No. 325, Section 2, Cheng-Kung Road, Neihu, Taipei 114, Taiwan; ^3^Department of Pathology, Kaohsiung Armed Forces General Hospital, Kaohsiung 80284, Taiwan; ^4^Department of General Surgery, Kaohsiung Armed Forces General Hospital, Kaohsiung 80284, Taiwan

## Abstract

We would like to highlight the application of natural products to hepatocellular carcinoma (HCC). We will focus on the natural products known as flavonoids, which target this disease at different stages of hepatocarcinogenesis. In spite of the use of chemotherapy and radiotherapy in treating HCC, patients with HCC still face poor prognosis because of the nature of multidrug resistance and toxicity derived from chemotherapy and radiotherapy. Flavonoids can be found in many vegetables, fruits, and herbal medicines that exert their different anticancer effects via different intracellular signaling pathways and serve as antioxidants. In this review, we will discuss seven common flavonoids that exert different biological effects against HCC via different pathways.

## 1. Introduction

### 1.1. Hepatocellular Carcinoma and Current Treatment

Hepatocellular carcinoma (HCC) accounts for approximately 5.6% of all tumors [1]. More than 80% of patients with liver cancer are diagnosed with HCC [2], which is resistant to most conventional therapies, such as chemotherapy and radiotherapy. Although HCC is the sixth most common neoplasm worldwide, its grave prognosis makes it the third leading cause of cancer-related mortality, responsible for approximately 600,000 deaths annually [3–5]. Hepatitis B and hepatitis C viruses are responsible for the highest frequencies of HCC occurrence in endemic areas, such as sub-Saharan Africa and far eastern Asia. In addition to these infections, alcohol consumption and exposure to dietary carcinogens, including aflatoxin B1 and nitrosamines, are other widely recognized etiological agents of HCC [6]. Carcinogenesis usually occurs as a result of chemical or biological damage to normal cells in a multistep and multifactor process composed of genetic derangement, aberrant signal transduction, and protein kinase activation. These processes contribute to the three stages of cancer development, which are known as initiation, promotion, and progression. Among the three stages, the promotion stage is reversible and appears to be the most appropriate target stage for chemopreventive intervention [7].

Hepatocarcinogenesis usually takes place under circumstances that include chronic viral infection or inflammation with further regeneration and cirrhosis. Influences, such as oxidative stress, which are caused by free radicals and immune responses, may contribute to DNA damage [8–11]. Current therapies targeting HCC, such as chemotherapy, surgical resection, transcatheter arterial embolization, percutaneous ablation therapy, liver transplant, and target therapy, do not always lead to optimal patient outcomes. Only surgery offers a cure, but tumor resection is feasible for <15% of patients. Even among those treated with curative intent, relapse rates are up to 50% [12]. HCC is often undetectable until late in the progression of the disease, and chemotherapy in the terminal stage does not usually exert much influence. Chemotherapy also has undesirable side effects, particularly in normal tissues with potent proliferative activity. Chemotherapy also adds little to the overall survival of patients with HCC because of the lower sensitivity of HCC cells, the emergence of drug resistance, and insufficient chemotherapeutic doses due to impaired liver function. Likewise, hepatectomy and liver transplantation can be curative, but high recurrence rates and subsequent low survival rates are still being reported [1, 2, 10, 12]. Palliative treatments associated with survival benefits include transcatheter arterial chemoembolization and sorafenib treatment. However, there is no consensus regarding the optimal treatment strategy [12].

Therefore, more effective therapeutic agents for treating HCC are desired, particularly specific agents that can target tumor cells instead of normal cells. The development of drugs that specifically target tumor cells or possess synergy with previous chemotherapeutic agents represents a common goal in drug development. Several clinical trials for HCC have been carried out with various therapeutic targets [13]. The aim of this review was to give an overview of the research in the field of flavonoids in HCC treatment and not only address the underlying mechanism of each flavonoid reported from several animal studies* in vitro* and* in vivo* on hepatoma cell lines [14–31] but also update its chemopreventive role on various targets [15, 22, 25, 32–43].

### 1.2. HCC and Flavonoids

Novel trends for comprehensive HCC therapy include chemoprevention induced by nature products, which is still under academic investigation. Chemoprevention is one of the strategies by which we can revert, suppress, or delay the response to carcinogens or even prevent carcinogenesis via the use of natural or synthetic chemical agents. Cancer chemopreventive agents are able to reduce the incidence of tumorigenesis by intervening in one or more of the stages of carcinogenesis: initiation, promotion, or progression. Many chemopreventive agents are derived from natural products, Chinese herbal medicines, and phytochemicals, namely, nonnutritive plant chemicals with protective or disease-preventive properties [44].

Natural products are nontoxic natural extracts or compounds that, compared with synthetic materials, generally produce fewer side effects; thus, they potentially provide an alternative or adjunct therapeutic option for patients with cancer.

In developing countries, approximately 35% of the prescribed drugs are derived from natural products, and over 60% of the anticancer drugs in clinical use originated from natural products [45], including plants, marine organisms, and microbes. Many investigations are being carried out worldwide to discover naturally occurring compounds that can suppress or prevent the progress of carcinogenesis, particularly in the field of HCC [46, 47]. The anticancer effects of most natural products often do not kill cancer cells directly. Rather, by regulating human immune function, they target the promotion stage by means of interrupting the cell cycle. This leads to the induction of apoptosis and differentiation, as well as the inhibition of cell growth and proliferation. These therapies may provide a second chance for the patient with terminal HCC and decrease HCC incidence in a preventive way. Recently, there has been a sustained growth of newly developed, effective compounds from natural products. The most often identified is the flavonoid family. The flavonoids are a group of plant secondary metabolites that comprise diverse classes of polyphenolic compounds found in fruits, vegetables, roots, stems, flowers, and beans, as well as beverages such as tea and wine, which can be ingested daily. These natural products were known for their beneficial effects on health long before flavonoids were isolated as the effective compounds. Attending to their molecular structures, flavonoids are usually divided into seven classes, including flavonols, flavanones, isoflavones, flavonols flavonolignans, and anthocyanidins. At the moment, more than 6,000 different natural flavonoids have been identified. Several studies have demonstrated that the increased taking of flavonoids is associated with several health-promoting properties, including a decreased risk of inflammation [48], hypertension [49], cardiovascular disease [50], allergic disease [51], and osteoporosis [52]. Some studies have also pointed out their antiviral [53–57] and antimicrobial properties [58, 59].

Studies of dietary flavonoids have revealed a broad spectrum of biological activities for these molecules, including the inhibition of cell proliferation in cell culture [46], induction of apoptosis [60], alteration of the activity of certain intracellular enzymes, and antioxidant properties [61]. Flavonoids are famous for their anticancer properties through multifactorial pathways [47]. The chemopreventive and chemotherapeutic effects of the flavonoids have been studied, and the most common effect of flavonoids is the scavenging of oxygen-derived free radicals. Other mechanisms focus on targeting the cancer promotion stage, the intracellular apoptotic pathway, and the cancer proliferation stage, including angiogenesis and metastasis [62, 63]. Natural compounds have been investigated for anticancer activities against lung cancer [64], breast cancer [65], gastric cancer and esophageal cancer [66], hepatocellular cell carcinoma [14, 67], colon cancer [68], prostate cancer [69], ovarian cancer [70], human cervical carcinoma [71], and leukemia [72]. In recent years, various flavonoids have been recognized as having potential protective activity against artificially induced-liver damage [24, 73, 74]. Accordingly, several studies have been conducted to investigate their chemopreventive abilities in HCC using various* in vitro* and* in vivo* methods [17–19, 25, 30, 34, 37, 43, 75–83].

## 2. Methods

**Figure 1:**
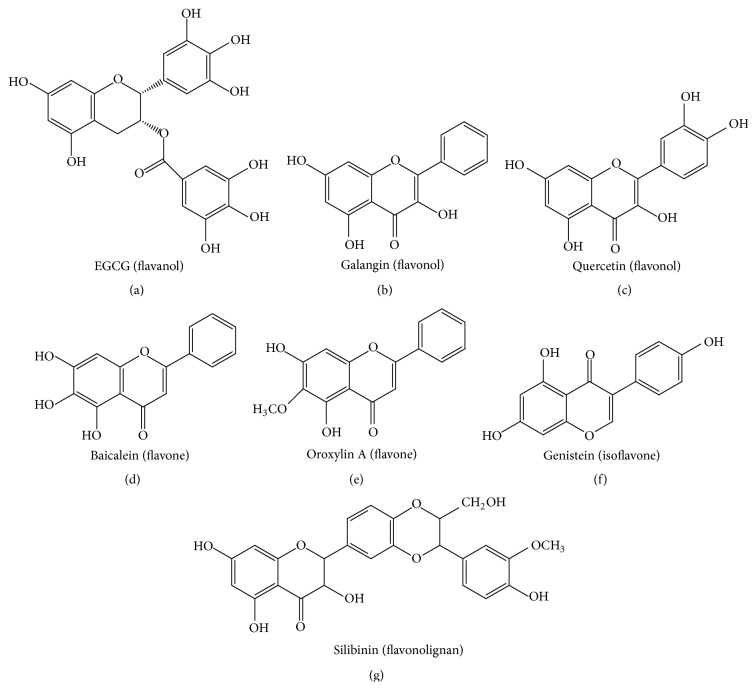
Chemical structures of flavonoids that target HCC: EGCG (a), galangin (b), quercetin (c), baicalein (d), oroxylin A (e), genistein (f), and silibinin (g). EGCG: (−)-epigallocatechin-3-gallate; HCC: hepatocellular carcinoma.

**Figure 2:**
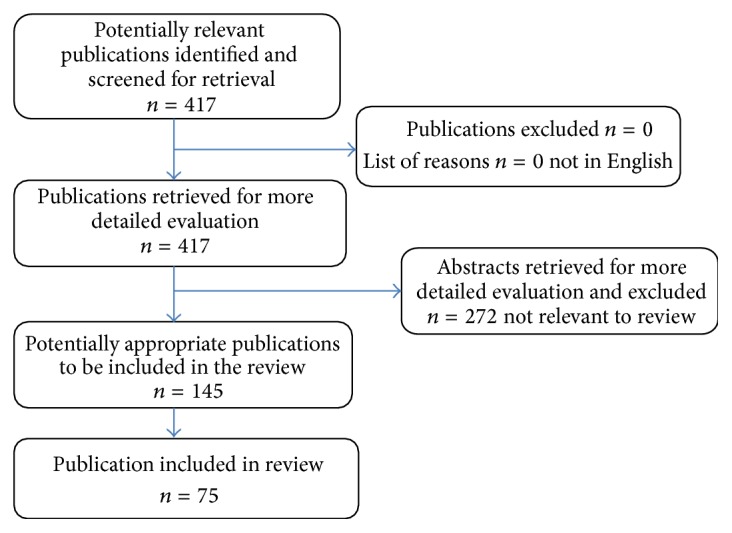
QUORUM algorithm of review of the flavonoids and hepatocellular carcinoma publications and abstracts.

In this paper, for the sake of clarity, we have researched the most common flavonoids that can affect HCC. Several reviews have provided major contributions to the current state of knowledge concerning the use of flavonoids for the treatment of liver cancer. The reports presented in the English language are reviewed to provide the full picture of the progression of the use of flavonoids against HCC. English literature searches were conducted through the Medline, Embase, Science Citation Index, Current Contents, and PubMed databases, as well as relevant articles from integrative and complementary medicine journals, such as* Evidence Based Complementary and Alternative Medicine*, until August 2015. After completing the searches of these English resources, the well-known flavonoids could be subclassified according to their structures. The seven common subclasses that have an effect on HCC are listed as flavanols [(−)-epi-gallocatechin-3-gallate (EGCG)], flavanones (liquiritigenin, hesperidin, naringenin, and xanthohumol), flavones (apigenin, baicalein, catechin, casticin, chrysin, diosmin, nobiletin, luteolin, oroxylin A, and wogonin), isoflavones (daidzein, equol, genistein, glabridin, puerarin, and tectorigenin), flavonols (fisetin, galangin, icariin, myricetin, kurarinol, and quercetin), anthocyanins, and flavonolignans (silibinin). Here we summarize the most common flavonoids, including EGCG, baicalein, oroxylin A, genistein, galangin, quercetin, and silibinin (Figure 1). The outline of the articles reviewed is presented in a Quality of Reporting of Meta-Analysis flow chart showing the number of studies screened and included in the meta-analysis (Figure 2) [84].

The search terms were “EGCG,” “galangin,” “quercetin,” “baicalein,” “oroxylin A,” “genistein,” “silibinin,” “hepatocellular carcinoma,” and “hepatoma.” Restrictions were placed on the language of publication, and only articles in English were included. Studies lacking controls and case reports were excluded.

The exclusion and inclusion criteria for data extracted from the literature were the same as those in PubMed. These restrictions were put in place to have consistency among the reports reviewed. The aim of this review was to give an overview of research in the field of flavonoids in HCC treatment, not only to address the underlying mechanism of each flavonoid in several animal studies and hepatoma cell lines but also to update their chemopreventive roles on various targets.

**Figure 3:**
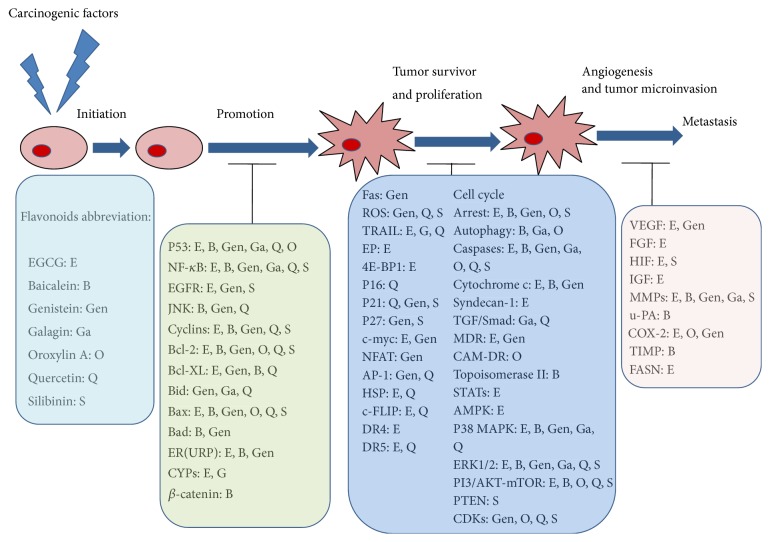
The different stages of HCC and the major targets modulated by various flavonoids. HCC: hepatocellular carcinoma; EGCG: (−)-epigallocatechin-3-gallate.

Typical signal pathways targeted during HCC treatment via the above flavonoids will be reviewed below. The corresponding signal pathways and flavonoids are mapped in Figure 3 and summarized in Tables 1 [Table tab2] [Table tab3] [Table tab4] [Table tab5] [Table tab6]–7, respectively.

### 2.1. (−)-Epi-gallocatechin-3-gallate

Tea is the most widely consumed beverage worldwide; furthermore, green tea is sold on a large scale, in part, due to its chemotherapeutic value [62, 63]. A typical cup of green tea generally contains 100–150 mg of catechins, including 50% (−)-epigallocatechin-3-gallate (EGCG), 15% EGC, 15% (−)-epicatechin-3-gallate, and 8% (−)-epicatechin [60]. More detailed,* in vivo* mouse studies have established that EGCG can function as a strong chemopreventive agent against cancer development and progression [115]. Numerous studies have shown that the green tea extract derived from the dried fresh leaves of the plant* Camellia sinensis* and one of its major constituents, EGCG, possess obvious antioxidant [116], antiproliferative [117], antiangiogenic [118], antimetastatic [119], and proapoptotic [120, 121] activities, as well as the ability to perturb the cell cycle [122] in various* in vitro* and* in vivo* tumor models. EGCG has immense potential as a therapeutic agent for the treatment and/or prevention of cancer due to its low cost and high bioavailability [123].

The anticancer role of EGCG has been investigated epidemiologically, in* in vitro* and* in vivo* models, as well as in clinical trials [124]. As the most potent antitumor component in green tea, EGCG has been shown to inhibit several critical signal transduction pathways [125], particularly the specific receptor tyrosine kinases and their downstream signaling pathways, in several cancer cell lines [126–128]. It has also been shown to have antiangiogenesis [129, 130] and antimetastatic properties [131, 132].

Particularly the investigation of critical signal transduction pathways in HCC has been carried out recently. The insulin-like growth factor (IGF) signaling axis has been shown to play a critical role in the development and progression of various tumors. The type 1 insulin-like growth factor receptor (IGF-1R) belongs to the tyrosine kinase receptor family. When activated by its ligands, it protects several cell types from a variety of apoptotic injuries. The main signaling pathway for IGF-1R-mediated protection from apoptosis has been previously elucidated and rests on the activation of phosphatidylinositol 3-kinase, Akt/protein kinase B, and the phosphorylation and inactivation of BAD, a member of the Bcl-2 family of proteins [133]. A recent study of the effects of EGCG on hepatoma cell lines has shown that EGCG inhibits the activation of IGF/IGF-1R-dependent signaling pathways by inducing apoptosis and causing a decrease in p-IGF-1R protein and its downstream signaling molecules, including the p-ERK, p-Akt, p-Stat3, and p-GSK-3*β* proteins [32]. The phosphatidylinositol 3′-kinase (PI3K)/AKT pathway has been reported to play an important role in cancer proliferation and migration. Previous studies indicate the role of the PI3K/AKT pathway in the development and progression of HCC cells, mainly reflected in the mechanism of liver cancer cell proliferation, differentiation, and apoptosis. EGCG affects both upstream and downstream targets of AKT [33]. Signal transducer and activator of transcription 3 (STAT3) is one of the seven members of the Stat protein family, which mediates the actions of many cytokines and growth factors. STAT3 serves as an oncogene that promotes cell survival, proliferation, and motility. STAT3 shows constitutive activity in many different types of cancers, including breast, prostate, head and neck, lung, colon, liver, and pancreatic cancers, as well as large granular lymphocytic leukemia and multiple myeloma [134]. A study has revealed that EGCG can inhibit HCC via the STAT3 pathway [86]. Recent studies indicate that AMPK activation strongly suppresses cell proliferation in nonmalignant cells, as well as in tumor cells. AMPK mediates these effects through multiple mechanisms, including regulation of the cell cycle, apoptosis, and autophagy, as well as the inhibition of protein synthesis and* de novo* fatty acid synthesis. Altered levels of AMPK have been linked with many human diseases, including cancer, and the modulation of AMPK has emerged as an important target for the treatment of obesity, diabetes, and cancer [135]. The AMPK signaling system contains some tumor suppressing genes, including L*κ*B1, TSC1, TSC2, and p53, and suppresses tumor growth by inhibiting the activity of various proto-oncogenes, such as PI3K, Akt, and ERK. The AMPK pathway is linked to tumor growth and proliferation through the inhibition of the mammalian target of rapamycin (mTOR) pathway via activation of the TSC2-TSC1 complex. EGCG has also been shown to be involved in the activation of AMPK via the suppression of downstream substrates, such as mTOR and eukaryotic initiation factor 4E-binding protein-1 (4E-BP1), as well as a general decrease in mRNA translation, which resulted in the apoptosis of p53-negative Hep 3B cells [88]. Proteinase-activated receptors (PARs) comprise a subfamily of G-protein-coupled receptors. PAR1, PAR3, and PAR4 are activated mainly by thrombin. Recently, PAR1 and PAR4 have been found to coordinately regulate HCC cell migration, suggesting a role for these two PAR subtypes in HCC progression [136]. Previous reports have shown that EGCG inhibits the thrombin-PAR1/PAR4-p42/p44 mitogen-activated protein kinase (MAPK) invasive signaling axis in HCC cells [15]. The apoptosis pathways have been ranked as the most common gate for anticarcinogenesis, which can be subclassified as the mitochondria-mediated intrinsic pathway, the death receptor-induced extrinsic pathway, and apoptotic signaling evoked by endoplasmic reticulum (ER) stress. Caspases are intracellular cysteine protease responsible for and associated with these three apoptosis pathways. The intrinsic apoptotic pathway is a mitochondria-involved signaling cascade in which caspase-9 is the predominant initiator caspase. The cytochrome *c* released from the mitochondria to the cytosol binds to Apaf-1, resulting in proteolytic processing and activation of caspase-9. Active caspase-9 then activates caspase-3, initiating a cascade of additional caspase activations that culminates in apoptosis. Caspase activation is regulated by various cellular proteins, including inhibitor-of-apoptosis, cellular FLICE-inhibitory protein (c-FLIP), and Bcl-2 family proteins. The Bcl-2 family of proteins can be divided into two groups: suppressors of apoptosis (e.g., Bcl-2, Bcl-XL, and Mcl-1) and activators of apoptosis (e.g., Bax, Bok, Hrk, and Bad). Some physiological proapoptotic molecules (e.g., Bax) are downregulated or inactivated in HCC, while many cancer cells resist apoptosis through upregulation of the Bcl-2 gene [137]. EGCG was shown to delay HCC cell growth through the inhibition of Bcl-2 family members [34, 75] or by inducing apoptosis in HCC cells via downregulation of COX-2 and Bcl-2 and consequently activating caspase-9 and caspase-3 [16]. In contrast to the intrinsic pathway, the extrinsic apoptotic pathway is mediated by death receptors, such as the receptors for Fas ligand (Fas-L) and tumor necrosis factor- (TNF-) related apoptosis-inducing ligand (TRAIL). Caspase-8 is a major initiator caspase in this pathway. Similar to the Fas/Fas-L system, TRAIL transduces apoptosis in a number of cancers. TRAIL, a member of the TNF family, is considered to be a promising anticancer agent due to its ability to induce apoptosis in a variety of tumor cell types. Cellular sensitivity to TRAIL depends on the expression of cell-membrane TRAIL receptors and caspase-8. Caspase-8 is activated in response to TRAIL and is released into the cytoplasm, where it initiates a protease cascade that activates effector caspases such as caspase-3 and caspase-7. TRAIL is known to trigger apoptosis through binding to death receptors DR4 and DR5. The subsequent interaction of DR4 or DR5 with the adaptor molecule FADD, via their respective death domains leads to recruitment and activation of caspase-8. Finally, caspase-8 activates the executioner caspases (e.g., caspase-3 and caspase-7), leading to apoptotic cell death. HCC is insensitive towards TRAIL-mediated apoptosis, suggesting that the presence of mediators can inhibit the TRAIL cell-death-inducing pathway in HCC. The combination of TRAIL with chemotherapeutic agents or anticancer cytokines may be a novel strategy for the treatment of HCC [138]. EGCG has been found to be capable of sensitizing cells toward TRAIL-induced apoptosis by upregulating of caspase-3 activity and DR4 and DR5 expression, downregulating of Bcl-2 expression, and decreasing the c-FLIP expression level [85].

In a study by Shan et al., a low concentration of acrylamide was able to significantly induce CYP2E1 expression in Hep G2 cells. EGCG was proposed to effectively reduce acrylamide-induced proliferation, as well as the expression of CYP2E1, epidermal growth factor receptor (EGFR), cyclin D1, and nuclear factor kappa-light-chain-enhancer of activated B cells (NF-*κ*B) proteins [17]. The ER is also a major organelle for oxygen and nutrient sensing as cells adapt to their microenvironment. Stresses that disrupt ER function lead to the accumulation of unfolded proteins in the ER, a condition known as ER stress. To prevent cells from the harmful effects of ER stress, a sophisticated signal pathway called the unfolded protein response (UPR) is triggered to attenuate protein translation, degrade misfolded protein, and induce molecular chaperone expression. A series of ER-localized proteins and enzymes, such as PKR-like ER kinase, inositol-requiring enzyme 1, and activating transcription factor 6 (ATF6), are activated by the UPR. CHOP, a downstream protein of ATF4 and ATF6, is the best characterized factor in the transition of ER stress to apoptosis. During UPR, the accumulated misfolded/unfolded proteins are subjected to protein degradation by the ubiquitin-proteasome pathway. However, if cells are unable to restore homeostasis because of prolonged ER stress, apoptosis is triggered in the damaged cells [139].

Normally, the UPR is considered a cell survival mechanism when ER stress is activated in many cell types. However, if ER stress persists, the proliferation of cells is inhibited. Because of the dual-cell survival/cell death roles of ER stress, this pathway remains a complicated target for cancer therapy. Therefore, a careful analysis of circumstances is required to determine whether to inhibit or promote ER stress for successful anticancer therapy. Emerging evidence suggests that the ER also regulates apoptosis both by sensitizing mitochondria to a variety of extrinsic and intrinsic death stimuli and by initiating cell death signals of its own. In addition to propagating death-inducing stress signals itself, the ER also contributes in a fundamental way to Fas-mediated apoptosis and to p53-dependent pathways resulting from DNA damage and oncogene expression. Mobilization of ER calcium stores can initiate the activation of cytoplasmic death pathways, as well as sensitize mitochondria to direct proapoptotic stimuli. Additionally, the existence of Bcl-2-regulated initiator procaspase activation complexes at the ER membrane has also been described [139]. Experiments carried out in microsomes and hepatoma cells revealed that EGCG can reduce the reactivation of carcinogens by inhibiting glucuronide transport in the ER and triggering ER stress and apoptosis in hepatoma cells by inhibition of glucosidase II [140]. The transforming growth factor-*β* (TGF-*β*) signaling pathway is believed to contribute to carcinoma development by increasing cell invasiveness and metastasis and inducing the epithelial-to-mesenchymal transition (EMT). TGF-*β* signaling occurs following the binding of the TGF-*β* ligand to TGF-*β* receptor I (TGF-*β* RI), which heterodimerizes with TGF-*β* RII. This heterodimer complex phosphorylates the intracellular proteins Smad2 and 3, activating a downstream cascade that produces a nuclear transduction protein. TGF-*β* is an important pathophysiological pathway in the liver associated with fibrogenesis. It promotes extracellular matrix (ECM) deposition in hepatic stellate cells after viral or metabolic injury. The final outcome of this process is decreased liver function, which often presents clinically as liver cirrhosis. This loss of liver function commonly precedes the onset of HCC in Western countries [141]. EGCG was found to downregulate the mRNA level of Smad7 and to mediate apoptosis in HepG2 cells via the TGF/Smad signaling pathway [35]. PGE2 is a prostaglandin that is abundant in HCC. Studies have established the important role of the PGE2 synthesis pathway as a potential target for the treatment and/or prevention of HCC [142, 143]. PGE2 exerts its biological activities primarily via G-protein-coupled prostaglandin receptors (four different receptors designated EP1, EP2, EP3, and EP4), which belong to the highly conserved superfamily of 7-transmembrane-spanning proteins [144]. Among these four EP receptors, studies have shown EP1 to be the most important in tumor development. EP1, through activation of EGFR/c-Met signaling, plays an important role in tumor cell invasion. A selective EP1 agonist increased the phosphorylation of EGFR, which suggests that it may enhance the invasion of tumor cells [145]. EGCG has also been shown to suppress EP1 receptor expression and PGE2 production [87]. EGCG can also influence HCC angiogenesis and metastasis via different mechanisms. EGCG was demonstrated to exert its antiangiogenesis ability through activation of vascular endothelial growth factor receptor- (VEGFR-) 2 and related downstream signaling molecules, including ERK and Akt [89]. EGCG also causes a drastic decrease in VEGF expression at both the mRNA and protein levels and inhibits the expression of serum-induced hypoxia-inducible factor 1 (HIF-1) alpha protein by interfering with the phosphatidylinositol 3-kinase/Akt/mTOR signaling pathways [92]. EGCG was found to exert its antimetastatic properties via inhibition of MMP-2 and MMP-9 activities [18, 19, 91, 146]. An additional study pointed out that EGCG inhibits vascular invasion via reduced expression of MMP-9, syndecan-1, and FGF-2 [36]. EGCG was also been found to exert its anticancer ability by reversing multidrug resistance [90].

### 2.2. Baicalein

Baicalein is one of the key flavonoids isolated from the roots of* Scutellaria baicalensis* or* Scutellariae radix* (Huangqin in Chinese), a famous Chinese medicinal herb. It possesses various biological activities, including anti-inflammatory [147], antioxidative [148], neuroprotective [149], cardiovascular protective [150], and antihyperglycemic [151] effects. It has broadly been used in remedies for hepatitis [152], cirrhosis [153], jaundice, and HCC in traditional Chinese, Japanese, and Korean medicine [96]. Accumulated evidence has demonstrated the anticancer potential of baicalein in plenty of cancer cell lines, including esophageal carcinoma [154], HCC [96], bladder cancer [155], prostate cancer [69], colon cancer [156], and pancreatic cancer [157]. The mechanisms underlying these baicalein-mediated anticancer effects include arrest of cancer cell cycle progression, induction of apoptosis, and blockage of invasion. Recent studies have shed light on the potential molecular pathways involved in the activity of baicalein against HCC [21, 37, 96]. Chang et al. had shown that baicalein induces cell cycle arrest and apoptosis in HCC cells. Their later study indicated that apoptosis induced by baicalein may be attributed to mitochondrial dysfunction [38]. Baicalein was found to trigger apoptotic episodes via the mitochondria-dependent caspase pathway, through activation of caspase-9 and caspase-3; increased Bax/Bcl-2 ratio and altered mitochondrial membrane potential with subsequent cytochrome *c* release; and the caspase-independent pathway via apoptosis-inducing factor (AIF) and endonuclease G (Endo G) release from mitochondria [20, 95, 158]. Autophagy is a dynamic degradation process for delivering dysfunctional cellular components or foreign invaders to lysosome to be digested by lysosomal hydrolase. Autophagy may inhibit tumor formation by degrading damaged organelles or proteins. After tumor formation, however, the tumor can use autophagy as a survival mechanism to counter against hypoxia, starvation, and an acid environment. Natural products as autophagy regulators have been reported to be prosurvival or prodeath [158]. A study by Wang et al. demonstrated that baicalein can trigger autophagy and inhibit the protein kinase B/mTOR pathway in a HepG2 cell line [20]. The c-Jun NH2-terminal kinase (JNK) signal transduction pathway induces defense mechanisms that protect organisms against acute oxidative and xenobiotic insults. This pathway has also been repeatedly linked to the molecular events involved in the regulation of autophagy [159]. Besides protective autophagy, baicalein also induced apoptosis via ER stress by downregulating prosurvival Bcl-2 family members, increasing intracellular calcium, and activating JNK [93]. The antitumor effect of baicalein may also be attributed to the deactivation of the PI3K/Akt pathway. A recent study from Zheng et al. demonstrated that baicalein inhibits Akt and promotes the degradation of *β*-catenin and cyclin D1 independent of GSK-3*β*. This result was also confirmed in an animal model [21]. Liang et al. recently revealed that MEK/ERK plays an important role both* in vitro* and* in vivo*. Baicalein inhibits MEK1 and subsequently reduces the activation of ERK1/2, leading to apoptosis and tumor growth arrest in mice with liver cancer [76]. Suppression of this pathway may also lead to attenuated cell migration and invasion by blocking multiple proteases that degrade the ECM [37]. NF-*κ*B is a ubiquitous transcription factor consisting of p50, p65, and I-kappa-B(I*κ*B)-*α*, which resides in the cytoplasm and is activated in response to various inflammatory stimuli, environmental pollutants, prooxidants, carcinogens, stress, and growth factors. Growing evidence shows that NF-*κ*B is one of the major culprits involved in the development of drug resistance in cancer, including HCC [160, 161]. Baicalein has been proposed to be involved in the NF-*κ*B pathway via decreasing p50 and p65 nuclear translocation and decreasing the phosphorylation of I*κ*B-*β* in hepatoma cells [94].

Metastasis is one of the leading causes of cancer-related death among patients with HCC. Cancer invasion and metastasis are complicated multistep processes involving numerous effector molecules. Degradation of the ECM in blood or lymph vessels is an essential step in cancer invasion and metastasis because loss of the ECM allows cancer cells to invade the blood or lymphatic system and spread to other tissues and organs. Matrix metalloproteinases (MMPs), a family of zinc-dependent endopeptidases that degrade almost all ECM components, play important roles in cancer invasion and metastasis.

Among the MMPs, MMP-2 and MMP-9 have been implicated in HCC invasion and metastasis. Upregulation of MMPs would facilitate degradation of the ECM, thus enhancing the metastatic ability of cancer cells [162]. In the plasminogen activation system, urokinase-type plasminogen activator (u-PA) activity may be the most sensitive factor reflecting HCC invasion; it is frequently used as a strong predictor of HCC recurrence [163]. Baicalein has been found to modulate antimetastatic effects and anti-invasion capabilities via inhibition of MMP-2 and MMP-9 activities [37]. The study demonstrated that treatment of HCC cell lines with baicalein decreases the expression of MMP-2, MMP-9, and u-PA, as well as tissue inhibitor of metalloproteinase-1 (TIMP-1) and TIMP-2, in a dose-dependent fashion [37, 94].

### 2.3. Genistein

Genistein (5,7,4′-trihydroxyisoflavone), a soybean-derived isoflavone, has been identified as a potential cause for the low incidence of certain types of tumors, including lung cancer [164], breast cancer [165], gastric cancer [166], colon cancer [167], HCC [168], and prostate cancers [169]. Genistein can exert its chemopreventive activities in each stage of multistep carcinogenesis through inhibiting tumor cell proliferation, inducing tumor cell differentiation, and triggering cell cycle arrest and apoptosis in some cell types [170, 171]. Genistein may affect HCC progression as a result of its effects on cell cycle progression and apoptosis [172]. Fang et al. demonstrated that genistein not only activated the mitochondrial apoptosis pathway through the activation of caspase-9 and caspase-3 followed by cleavage of poly (ADP-ribose) polymerase (PARP) but also activated the Fas pathway by increasing the expression of Fas, FasL, and p53 proteins [100]. Genistein has also been demonstrated to increase the production of intracellular reactive oxygen species (ROS) and has been found to exert a synergistic effect with arsenic trioxide (ATO) via the mitochondrial apoptosis pathway [101]. Notably, it has been suggested that ER stress and the associated activation of NF-*κ*B, ATF-6, and MAPKs may contribute to hepatocarcinogenesis. A study by Yeh et al. demonstrated that genistein induces the activation of several ER stress-relevant regulators, including m-calpain, GADD153, GRP78, and caspase-12 [77]. Activation of caspase-2, manifested as mitochondrial insult by genistein, also triggers apoptotic cell death [77]. Genistein has been reported to suppress ATK and the NF-*κ*B pathway through different pathways [98, 99]. Combination of ATO with genistein presents a promising therapeutic approach for the treatment of HCC. Its efficacy is mediated via the suppressive effect of genistein on both proper and ATO-induced Akt activation and on the activity of NF-*κ*B. The latter correlates with the suppression of NF-*κ*B-regulated gene products, including cyclin D1, Bcl-xL, Bcl-2, c-myc, COX-2, and VEGF [99]. The generic MAPK signaling pathway is shared by four distinct cascades, including the extracellular signal-related kinases (ERK1/2), the Jun aminoterminal kinases (JNK1/2/3), p38-MAPK, and ERK5. The MAPK/ERK pathway is reported to be associated with the cell proliferation, differentiation, migration, senescence, and apoptosis [173]. Jin et al. also reported that genistein enhances TRAIL-induced apoptosis through inhibition of p38 MAPK signaling in human HCC Hep3B cells [39]. Genistein possesses anti-invasive and antimetastatic activities against TPA-mediated metastasis via downregulation of MMP-9 and EGFR and subsequent suppression of NF-*κ*B and AP-1 transcription factors through the inhibition of MAPK, I*κ*B, and PI3K/Akt signaling pathways [98].

The EMT is of paramount relevance for embryonic development and adult wound healing. The EMT contributes to the formation and differentiation of tissues and organs. The conversion of epithelial cells to mesenchymal cells is critical for embryonic development and is implicated in phenotypic changes such as the loss of cell-cell adhesion, changes in cell polarity, and the acquisition of migratory properties. During the past decade, the EMT has been increasingly recognized to occur during the progression of various carcinomas, such as HCC. Hepatocellular EMT is a crucial event in HCC progression that causes an increase in hepatocyte malignancies associated with tumor cell invasion and metastasis [174]. In humans, the nuclear factor of activated T cells (NFAT) family comprises five subtypes, referred to as NFAT1–5. They are ubiquitously expressed in various mammalian tissues. The NFAT pathway axis is a vertebrate-specific signaling pathway important for different cellular functions. Several recent studies have demonstrated the important roles for NFATs in regulating phenotypes related with malignancy and neoplastic progression. NFATs are upregulated in human solid cancers and hematological tumors and appear to have functions in cancer cell-autonomous actions such as invasion, migration, and differentiation, as well as the proliferation of cells in tumors and its microenvironment. Dai et al. reported that treatment with genistein suppressed the EMT induced by TGF-*β*, which may also contribute to reduced NFAT1, and inhibited the intrahepatic metastasis [22]. Gu et al. reported that genistein had antimetastatic potential, with a proposed mechanism involving cell cycle progression and apoptosis in MHCC97-H cells both* in vitro* and* in vivo* [78].

### 2.4. Oroxylin A

Oroxylin A is a naturally occurring monoflavonoid extracted from the root of* S. baicalensis* Georgi or* S. radix*. Oroxylin A, which manifests as a major bioactive flavone and exists in the aglycone form. It is known to exert anti-inflammatory [175], antiviral [176], antiangiogenesis [55], antipruritic [177], and neuroprotective properties [178] and to restore liver function [179]. The anticancer effects of oroxylin A have also been studied in several cell lines, such as those derived from lung cancer [180], breast cancer [181], gastric cancer [182], HCC [24], colon cancer [183], cervical cancer [184], and leukemia [185].

Investigations of its activity against HCC have also been carried out recently. MDM2 was proposed to be related to the high invasiveness of HCC through inactivation of the tumor-suppressor function of the p53 gene [186]. Oroxylin A could serve as a novel candidate for cancer therapy through its stabilization of p53 expression, as well as its induction of apoptosis at the posttranslational level via the downregulation of MDM2 expression and its interference with MDM2-modulated, proteasome-related p53 degradation [187]. The mitochondrial apoptotic pathway has been investigated as the mechanism by which oroxylin A exerts its chemopreventive activity in HCC. Oroxylin A was found to effectively induce programmed cell death by suppressing expression of Bcl-2 protein and pro-caspase-3 protein and also by dramatically increasing in the number of cells undergoing apoptosis and G(2)/M phase arrest [97]. Oroxylin A was also noted for its synergistic effect when combined with 5-fluorouracil (5-FU) in human HCC HepG2 cells* in vitro* and in transplanted murine hepatoma 22 (H22) tumors* in vivo*. Oroxylin A enhanced 5-FU-induced apoptosis in HepG2 cells by elevating the expression of apoptosis-inducing proteins p53 and cleaved PARP and by decreasing expression of apoptotic-inhibitory proteins COX-2, Bcl-2, and pro-caspase-3 [79]. PTP and MAC are mitochondrial channels involved in the permeabilization of the mitochondrial outer membrane. MAC forms in the mitochondrial outer membrane early in apoptosis and provides a direct pathway for the release of cytochrome *c* from the intermembrane space to the cytosol. Liu et al. has suggested that opening of the MAC, but not PTP, plays a key role in oroxylin A-induced activation of the mitochondrial apoptotic pathway in HepG2 cells [40]. In addition to the mitochondrial apoptotic pathway, the unfolded protein pathway has also been reported to be an important chemoprevention mechanism for oroxylin A in HCC. Xu et al. proposed that an underlying molecular mechanism implicated the H_2_O_2_-triggered overactivation of the UPR pathway and causal inactivation of AKT signaling in the preferential cytotoxicity of oroxylin A in malignant HepG2 cells [23]. Oroxylin A also exerts its anticarcinogenic properties by targeting aberrant activation of antiapoptotic signaling pathways. Zou et al. demonstrated that oroxylin A exhibits autophagy-mediated antitumor activity in a dose- and time-dependent manner* in vivo* and* in vitro*, which is related to the suppression of the phosphatase and tensin homolog- (PTEN-) Akt-mTOR signaling pathway [24]. Integrin-mediated adhesion influences cell survival and may prevent programmed cell death. Oroxylin A can reverse the resistance caused by cell adhesion-mediated drug resistance (CAM-DR) via inhibition of integrin *β*1 and its related pathway, which can dramatically increase the apoptosis induced by paclitaxel in the CAM-DR model of HepG2 cells [80].

### 2.5. Galangin

Galangin is a flavonol, a type of flavonoid present in high concentrations in medicinal plants (e.g.,* Alpinia officinarum*), and propolis, a natural beehive product. Galangin has been shown to have antibacterial [188], antiviral [58], anti-inflammatory [56], antioxidant [189], antiallergic [190], and antiobesity [191] activities, as well as neuroprotective [192] and antineoplastic properties [193]. Galangin has potent anticancer activity against several cancer cells, such as lung cancer [194], head and neck cancer [195], breast cancer [196], HCC [25], colon cancer [197], ovarian cancer [198], leukemia [199], and melanoma cells [200]. The chemopreventive role of galangin arises through targets in different pathways. The study by Zhang et al. has shown that galangin induces apoptosis in HCCs by activating the caspase-8/t-Bid mitochondrial pathway [41, 104]. Su et al. have proposed that galangin induces ER stress via the MAPK pathway [103]. Galangin has been found to trigger apoptosis through the autophagy pathway. TGF-*β* differentially regulates autophagy in a context-specific manner. TGF-*β* induces the accumulation of autophagosomes and increases the degradation rate of long-lived proteins. TGF-*β* increased the mRNA expression levels of BECLIN1 (BECN1), ATG5, ATG7, and death-associated protein kinase (DAPK). Knockdown of Smad2/3, Smad4, or DAPK or inhibition of c-Jun NH(2)-terminal kinase attenuates TGF-*β*-induced autophagy, indicating the involvement of both Smad and non-Smad pathways [26]. Among the two opposing TGF-*β* Smad pathways in endothelial cell systems (Smad1/5/8 and Smad2/3), Smad2 was found to be the major transcriptional regulator of autophagy that targets BECN1 gene expression. Smad2, but not Smad3, acts as a repressor upstream of the BECN1 promoter region [201].

Wang et al. found that galangin induces autophagy via activation of the TGF-*β* receptor/Smad pathway; increases TGF-*β* RI, TGF-*β* RII, Smad1, Smad2, Smad3, and Smad4 levels but decreases Smad6 and Smad7 levels [25]; and also mediates autophagy via upregulation of p53 [81]. Chien et al. found that galangin inhibits the TPA-induced invasion and migration of HepG2 cells through a protein kinase C/ERK pathway, resulting in the suppression of MMP-2/MMP-9 enzymatic activity [202].

### 2.6. Quercetin

Quercetin is a flavonol found in abundance in onions, grapes, berries, and apples, as well as green vegetables such as broccoli and grains. It can be used as an ingredient in supplements and beverages (tea, wine, and beer) that have been shown to prevent a variety of human diseases due to their antihypertensive [203], anti-inflammatory [204], antioxidant [205], antimicrobial [206], antiviral [59], antiallergic [57], and antidiabetic [207] activities, as well as their neuroprotective [208] and anticancer effects [209]. Recent studies have found that quercetin can prevent or slow the progression of a wide variety of tumors, including lung cancer [210], gastric cancer [211], colon cancer [212], prostate cancer [213], ovarian cancer [214], and HCC [215]. Quercetin has antioxidant activity, particularly during the initiation of HCC carcinogenesis [216, 217]. Chang et al. has demonstrated that quercetin and 2-methoxyestradiol exhibit synergistic cytotoxic effects through increasing superoxide levels, annexin V binding, and mitochondrial disruption in HA22T/VGH and HepG2 cell lines [109]. Quercetin can also trigger cell cycle arrest. Quercetin can induce apoptotic cell death by regulating the cell cycle and suppressing antiapoptotic proteins such as Sp1 and Sp1 regulatory protein [27].

Li et al. proposed that quercetin-induced cell apoptosis involves induction of G2/M arrest, apoptosis, and cell death and is associated with increased expression of p53 and p21; decrease of cyclin D1, cyclin-dependent kinase (CDK) 2, and CDK7 levels; and generation of reactive oxygen species in cells [28]. Quercetin also has suppressive activity against HCC cells through p16-mediated cell cycle arrest and apoptosis; its combination with cisplatin yielded synergistic inhibitory effects in suppressing cell growth and inducing apoptosis [105]. Quercetin has been known to trigger apoptosis via the mitochondrial, Akt, and ERK pathway, as observed in quercetin-treated HepG2 cells [42, 106]. Quercetin was also found to induce a significant time-dependent inactivation of the NF-*κ*B pathway and a time-dependent activation of the AP-1/JNK pathway. Quercetin contributes to the regulation of survival/proliferation (AKT, ERK) and death (caspase-3, p38, and unbalance of Bcl-2 proapoptotic and antiapoptotic proteins) signals in a human hepatoma cell line (HepG2) [82, 108]. Quercetin was found to potentiate the antitumor effects of doxorubicin in liver cancer cells while protecting normal liver cells* in vitro* and* in vivo* with a proposed mechanism of p53-dependent downregulation of Bcl-xl expression [83]. c-FLIP is a master antiapoptotic regulator and resistance factor that suppresses TNF-*α*, Fas-L, and TRAIL-induced apoptosis, as well as apoptosis triggered by chemotherapy agents in malignant cells. c-FLIP binds to FADD and/or caspase-8 or caspase-10 and TRAIL receptor 5 (DR5) in a ligand-dependent and -independent fashion and forms an apoptosis inhibitory complex. This interaction, in turn, prevents formation of the death-inducing signaling complex and subsequent activation of the caspase cascade. c-FLIPs are also known to have multifunctional roles in various signaling pathways, as well as activating and/or upregulating several cytoprotective and prosurvival signaling proteins including Akt, ERK, and NF-*κ*B [218]. Quercetin has been shown to recover TRAIL sensitivity in various HCC cells via upregulation of DR5 (a death receptor of TRAIL) and downregulation of c-FLIPs [110]. Heat shock proteins (HSPs) consist of a large group of proteins with negligible expressions under physiological conditions. Their expression is highly induced under stress conditions, and they are ubiquitously expressed in various tissues and organs. HSPs possess chaperone functions, facilitating the correct folding of proteins or peptides. High expression of HSPs has been demonstrated in liver cancer tissues and is correlated clinically with the severity of tumors and poor outcomes in patients with HCC [219]. Zhou et al. demonstrated that quercetin can exert a significant inhibitory effect on overall expression of HSPs in HepG2 cells, which may be a novel pathway for anticarcinogenesis [107] Sharma et al. also reported that quercetin was able to potentiate the proapoptotic action of 5-FU and carboplatin by decreasing expression of HSPs such as Hsp40 and Hsp27 [220].

### 2.7. Silibinin

Silibinin is a polyphenolic mixture of flavonolignans and the major biologically active compound extracted from the seeds of milk thistle [*Silybum marianum* (L), Gaertn.]. It is used around the world, including the United States, Europe, and Asia, as a traditional herbal/dietary supplement because of its strong antihepatotoxic activity against almost any kind of human liver damage/toxicity [221, 222]. The crude form of milk thistle extract, silymarin, and the major pure pharmacologically active flavonoid, silibinin, which is composed of a 1 : 1 mixture of Silibinins A and B, has been shown to be immune-response modifiers* in vivo* [73]. It is well known that milk thistle is safe and well tolerated and that it protects the liver from drug or alcohol-related injury [74]. Thus, they have been available as over-the-counter drugs in European and Asia countries to protect the liver against external poisons. In terms of their efficacy against cancer, several studies have shown that both silymarin and silibinin are highly effective in the prevention and intervention of various cancers in both rodent and cell culture models and that their mechanisms of efficacy involve cell cycle arrest and/or apoptosis [223, 224]. The cancer sites showing silymarin and silibinin efficacy include prostate [225], skin [226], lung [227], breast [228], colon [229], HCC [29], and bladder [230].

Silibinin has been demonstrated to cause G1 arrest in HepG2 cells and both G1 and G2-M arrests in Hep3B cells. They exert their effects via Kip1/p27 but decrease cyclin D1, cyclin D3, cyclin E, CDK2, and CDK4 levels in both cell lines. Silibinin further strongly inhibits CDK2, CDK4, and CDC2 kinase activities in these HCC cells [114]. The PTEN/PI3K/Akt pathway has been associated with carcinogenesis [231]. PTEN is a negative regulator of PI3K-Akt signaling [232] and one of the most frequently inactivated genes in malignancies. Akt is a downstream protein kinase of PI3K (PTEN) and is a signal transduction protein that has been identified as one of the key elements in protecting cells from apoptosis. If unregulated, Akt promotes uncontrolled cell replication. The PI3K/Akt/mTOR pathway has been proposed to be correlated with silibinin-mediated anti-HCC effects [113]. In an* in vivo* study, Cui et al. also proposed that silibinin reduces HuH7 xenograft growth through the inhibition of cell proliferation, cell cycle progression, and PTEN/P-Akt and ERK signaling; induces cell apoptosis; and increases histone acetylation and SOD-1 expression [111]. Silibinin was also found to exhibit synergy with sorafenib or gefitinib, which is attributable to inhibition of EGFR-dependent Akt signaling [30]. HIF-1 plays a critical role for tumor adaptation to microenvironmental hypoxia and represents an appealing chemotherapeutic target. Inhibition of mTOR, a well-known regulator of cell cycle progression and cell proliferation, causes strong G1-phase cell cycle arrest. In addition, mTOR activity can regulate apoptotic cell death in some situations [233]. A study has shown that silibinin can exert its antiproliferative effect via inhibition of HIF-1*α* and the mTOR/p70S6 K/4E-BP1 signaling pathway in hepatoma cells [112]. Momeny et al. also demonstrated that silibinin inhibits cell growth and invasive properties in human HCC through inhibition of ERK 1/2 phosphorylation in HepG-2 cells [43]. The Notch signaling pathway plays a role in cell proliferation, differentiation, tumorigenesis, and immune development. A recent report proposed a close relationship between the Notch signaling pathway and HCC pathogenesis [234]. Silibinin can trigger apoptosis via inhibition of Notch signaling, upregulation of the apoptosis pathway-related protein Bax, and downregulation of Bcl-2, survivin, and cyclin D1 [29]. Silibinin also demonstrated antiangiogenic and antimetastatic activities. The antiangiogenic effect of silibinin has also been demonstrated via the downregulation of MMP-2 and CD34 [113]. Silibinin can potentially function as a multitargeting antimetastatic agent via suppression of signals at the transcriptional level and depression of the enzymatic activity of MMP-2 [235].

## 3. Conclusion

Surgical resection and liver transplantation are the first-line treatments for HCC. However, recurrence after surgery represents a tough problem, and the prognosis of patients with recurrent disease is pessimistic. For patients with advanced-stage HCC who lack the opportunity to receive curative therapy, effective treatment is even more limited. HCC is well known for its resistance to chemotherapy. Systemic chemotherapy using traditional cytotoxic drugs has little effect on patients with HCC. This leaves the small molecule targeted drug sorafenib, which targets VEGFR and is a tyrosine kinase inhibitor, as the only medication with evidence to improve the prognosis of patients with advanced-stage HCC. However, its unprecedented high cost and severe adverse effects remain obstacles for HCC treatment. The absence of an ideal therapy for HCC largely contributes to the current dilemma of HCC treatment. Flavonoids have recently been well studied for their anticancer properties and have been widely adopted due to their advantages of high efficiency, weak side effects, easy availability, and improvement of the quality of life. Several flavonoids are among the new era of evolutionary therapies that affect HCC through several pathways and target different stage of carcinogenesis. Flavonoids have recently been found to exert their anticancer properties through triggering cell cycle arrest; upregulating tumor suppressors, such as p53; interacting with various pathways, including the intrinsic (mitochondrial apoptosis pathway); and extrinsic (the death receptor pathway) apoptotic pathways, the ER-related unfolded protein pathway, autophagy, EGFR/c-Met signaling pathway, TRAIL-induced apoptosis signaling pathway, NF-*κ*B-related pathway, JAK/STAT pathway, HSP-related pathway, tumor suppressor-related pathway, MAPK/JNK-mediated pathway (also known as the Ras-Raf-MEK-ERK pathway), JNK pathway, PI3K-PTEN-Akt-mTOR signaling pathway, and TGF-*β* signaling pathway. They also target HCC angiogenesis and metastasis stages via multiple intracellular signals. It is still believed that a combination of drugs with different mechanisms of action may provide some benefit to overcome drug resistance and reduce side effects. Approximately 67% of all anticancer drugs originate from the bioactive compounds found in plants. Several flavonoids have been proposed to exert their synergistic effect through different mechanisms. In addition to their synergistic effects with current chemotherapeutic agents [79, 83, 105] or in combination with hormonal agents [109], combinations with two flavonoids have also shown synergistic effects in treating HCC. Chen et al. have demonstrated that the combination with baicalein and silymarin in human hepatoma HepG2 cells synergistically increased the percentage of cells in G0/G1 phase and decreased those in S-phase. These effects were associated with the upregulation of Rb, p53, p21, and p27 and the downregulation of cyclin D1, cyclin E, CDK4, and phospho-Rb, which offer mechanistic insight for their further exploitation in HCC treatment [102]. Several clinical trials have shown a dose-dependent relationship between flavonoids and their anticarcinogenic effects. However, overdose can lead to possible side effects. EGCG at high doses was reported to cause liver injury [236, 237]. Long-term toxicity also needs to be taken into account. At present, most of the anti-HCC candidates listed in this paper still lack sufficient data on their toxicity/side effects, although they have been frequently used in analyses* in vitro* and* in vivo*. Therefore, studies on their toxicity/side effects in both animals and humans should be a future direction. A randomized, double-blind, single-dose trial of baicalein conducted by Li et al. proved that single oral doses of 100–2,800 mg baicalein were safe and well tolerated by healthy subjects with no toxicity to the liver or kidney [238]. In spite of their anti-HCC actions in preclinical models, the low bioavailability of many potential candidates greatly limits their clinical efficacy.

Currently, silymarin, the crude form of mild thistle extract, has been successfully formulated into pills and broadly carried into the clinical fields against several hepatic diseases. A clinical study showed that short-term administration of Silibinin phosphatidylcholine (siliphos) in patients with advanced HCC resulted in detectable increases in silibinin and its metabolite, silibinin glucuronide. However, the trial failed to establish the maximum tolerated dose because the patient died soon after enrollment [239]. Silibinin has been demonstrated to have poor water solubility (approximately 40 *μ*g/mL) and oral bioavailability (approximately 0.73%) in both rodents and humans, which restrict its clinical application [240]. In recent decades, emerging nanotechnologies provide a novel platform to solve the drug solubility problem. Polymeric micelles have the ability to encapsulate a hydrophobic drug and deliver the drug to the desired site at a concentration exceeding the intrinsic solubility of the drug. Moreover, the encapsulated drug is not only protected from contact with the contents of the gastrointestinal tract, which likely induces degradation and metabolism but also conferred with the characteristics of sustained release and direct uptake by cells. Many studies have proven that nanoparticles can transport across the intestinal membrane through paracellular or transcellular routes [241]. Duan et al. have developed mixed micelles loaded with a Silibinin-polyene phosphatidylcholine complex to improve its drug solubility and prolong its mean retention time* in vivo* [242]. Quercetin has also been reported to have poor water solubility and poor oral bioavailability in rats (approximately 17%) and in humans (1%) [243]. Mandal has demonstrated that nanoencapsulated quercetin downregulates rat hepatic MMP-13 and controls diethylnitrosamine-induced hepatocarcinogenesis [244]. At present, quercetin is considered safe only based on* in vitro* studies [245]. Further* in vivo* evaluations and clinical trials are absolutely necessary to ascertain the maximum tolerated dose and the efficacy of quercetin in adjuvant HCC therapy. Nutrient-drug interactions have been recently emphasized. Although genistein has recently been shown to exert a synergistic effect with several chemotherapeutic agents [101, 246], Rigalli et al. recently demonstrated that ingestion of genistein-rich soy products or dietary supplements in combination with sorafenib can result in chemoresistance via induction of multidrug resistance-associated protein 2 [31]. In contrast, Gu et al. have demonstrated that combination treatments with silibinin enhance the growth-inhibitory effects of both gefitinib and sorafenib, which may be attributable to the inhibition of EGFR-dependent Akt signaling [30]. Hepatocarcinogenesis usually takes a long period after exposure of the noxious stimulus, which means the duration of intake of the flavonoid is a major concern for exerting its chemopreventive effect. Thus, compliance in this group of people is also of great importance. Patient selection for those at high risk for developing HCC is also of great significance. Flavonoids may exert different chemopreventive effects, alone or in combination, against diverse etiologies of HCC. This still needs to be investigated via clinical trials. Most of the studies in most of our reviews were* in vitro* studies within a limited number of hepatoma cell lines. Further* in vivo* evaluations and clinical trials are absolutely necessary to ascertain the maximum tolerated dose and the efficacy of flavonoids in adjuvant HCC therapy. We expect that future study can translate flavonoids into clinical medicine and shed light on novel treatments for the disastrous disease known as HCC.

## Figures and Tables

**Table 1:** EGCG in HCC with possible mechanisms discussed in this paper.

Subfamily	Flavonoids	Typical origin	*In vitro*/*in vivo* Cell lines/modes Effective dose	Effects and related mechanisms	Author (year) References
Flavonol	EGCG	Green tea red grapes and red wines	*In vitro*/*in vivo* HepG2/SD rats by thioacetamide 20 mg/kg/N/A	↓ *α*-FP restoration of heparin sulfate proteoglycans receptors, ↓ vascular invasion via ↓ MMP-9, ↓ syndecan-1 ↓ FGF-2, and ↓ fibrosis	Darweish et al. (2014) [36]

			*In-vitro* Acrylamide-induced HepG2 cells 10 *μ*M	↓ CYP2E1, ↓ EGFR, ↓ cyclin D1 and ↓ NF-*κ*B ↓ growth by affecting the cell cycle, and ↑ apoptosis	Shan et al. (2014) [17]

			*In vitro* HepG2, SMMC7721 and SK-hep1 cells IC50 74.7, 59.6, and 61.3 *μ*g/mL	↑ apoptosis by ↓ PI3K/AKT activity, ↓ NF-*κ*B, and ↑ S phase arrest (in SMMC7721 cells)	Shen et al. (2014) [33]

			*In vitro* HepG2 cells 70 *μ*g/mL	↑ TRAIL-induced apoptosis via ↑ caspase-3 activity, ↑ DR4 ↑ DR5 expression, and ↓ Bcl-2 expression and ↓ c-FLIP expression level	Abou El Naga et al. (2013) [85]

			*In vitro* BEL-7402 and QGY-7703 40 *μ*M	↓ STAT3 signaling pathway. ↓ Bcl-xL, ↓ c-Myc, ↓ VEGF, and ↓ cyclin D1	Wang et al. (2013) [86]

			*In vitro* HCCLM6 cells dose-dependent (10–100 *μ*g/mL)	↑ apoptosis, ↓ metastasis via ↓ MMP-2, ↓ MMP-9 ↓ HSP*β*1, and ↓ chaperonin	Zhang et al. (2013) [18]

			*In vitro* HepG2 cells 100 *μ*g/mL	↓ EP(1) receptor expression and ↓ PGE(2) production	Jin et al. (2012) [87]

			*In vitro* HepG2 cells 100 *μ*M	↑ apoptosis and ↓ Bcl-2 and ↑ miR-16	Tsang and Kwok (2010) [34]

			*In vitro* HEP-3B 10 *μ*M	↓ thrombin-induced HCC invasion and p42/p44-MAPKinase activation	Kaufmann et al. (2009) [15]

			*In vitro* p53 positive Hep G2 and p53 negative Hep 3B cells 80 *μ*M	↑ AMPK, ↓ mTOR, ↓ 4E-BP1, ↓ mRNA translation, ↓ FASN, and ↓ ACC	Huang et al. (2009) [88]

			*In vitro*/*in vivo* HuH7 cells/nude mouse xenograft model 25 *μ*g/mL, time dependent; 0–100 *μ*g/mL, dose dependent/0.01%–0.1%	↓ VEGF2, ↓ p-VEGFR-2; ↓ ERK and Akt, and ↓ Bcl-x(L)	Shirakami et al. (2009) [89]

			*In vitro* BEL7404/ADM cells, BEL7402/5-FU cells IC10 24.76 mg/L, 20.60 mg/L IC50 3.85 mg/L, 2833.62 *μ*mol/L	↓ MDR in HCC, ↓ MDR1, ↓ LRP expression, and ↑ cyclin G1 expression	Tang et al. (2008) [90]

			*In vitro* HepG2, SMMC-7721 100–400 *μ*g/mL	↓ COX-2, ↓ Bcl-2 (over 200 *μ*g/mL) ↑ caspase-9 and ↑ caspase-3 (100 and 200 *μ*g/mL for 12 h)	Chen et al. (2008) [16]

			*In vitro* HepG2 20 *μ*g/mL	↓ IGF/IGF-1 receptor dependent signaling pathway by ↑ apoptosis, ↓ p-IGF-1R protein, ↓ p-ERK, ↓ p-Akt, ↓ p-Stat-3, and ↓ p-GSK-3*β* proteins; ↓ IGF-1, IGF-2, and ↑ IGFBP-3	Shimizu et al. (2008) [32]

			*In vitro* SK-Hep-1 cells 20 *μ*g/mL	↓ tumor invasion and migration via ↓ MMP-9 and MMP-2	Sang et al. (2007) [91]

			*In vitro*/*in vivo* HLE cells/BALB/c nude mice 0–100 *μ*g/mL/0.8, 2.5 and 7.5 mg/mL	↑ apoptosis in HLE cells via ↓ Bcl-2*α*, ↓ Bcl-xl ↓ NF-*κ*B; ↑ TRAIL-induced apoptosis	Nishikawa et al. (2006) [75]

			*In vitro* HepG2 50 *μ*mol/L	↓ hypoxia induced HIF-1 protein; ↓ VEGF; ↓ PI3/Akt/mTOR pathways, and ↓ ERK 1/2 signaling pathways	Zhang et al. (2006) [92]

↑: Intensify; ↓: attenuate; N/A: none available; EGCG: (−)-epi-gallocatechin-3-gallate; SD rats: Sprague Dawley rats; DEN: diethylnitrosamine; MMP: matrix metalloproteinase; TIMP: metallopeptidase inhibitor 1; FGF: fibroblast growth factor; EGFR: epidermal growth factor receptor; CYP: cytochrome; PKC*α*: protein kinase C-alpha; EMT: epithelial-mesenchymal transition; I*κ*B-*β*: I-kappa-*β*; PI3: phosphatidylinositol 3-kinase; PTEN: phosphatase and tensin homolog; NFAT1: nuclear factor of activated T cells 1; DR: death receptor; TRAIL: tumor necrosis factor-related apoptosis inducing ligand; c-FLIP: cellular FLICE inhibitory protein; STAT3: signal transducer and activator of transcription 3; VEGF: vascular endothelial growth factor; EP(1): prostaglandin E receptor 1; MAPK: p38-beta mitogen-activated protein kinase; AMPK: AMP-activated protein kinase; mTOR: mammalian target of rapamycin; 4E-BP1: eukaryotic initiation factor 4E-binding protein-1; FASN: fatty acid synthase; ACC: acetyl-CoA carboxylase; ERK: extracellular signal regulated kinases; MDR: multidrug resistance; LRP: lung resistance protein; IGF: insulin-like growth factor; GSK-3*β*: glycogen synthase kinase-3*β*; HIF-1: hypoxia-inducible factors; IGFBP-3: insulin-like growth factor binding protein-3; ER: endoplasmic reticulum; UPR: unfolded protein response; JNK: c-Jun N-terminal kinases; MTP: mitochondrial transmembrane potential; CAM-DR: cell adhesion mediated drug resistance; MDR: multidrug resistance; MAC: mitochondrial apoptosis-induced channel; AIF: apoptosis-inducing factor; 5-FU: 5-fluororuracil; PARP: poly ADP-ribose polymerase; AP-1: activator protein 1; ATO: arsenic trioxide; FAK: focal adhesion kinase; CDK: cyclin-dependent kinases; GRP: glucose-regulated protein; CHOP: C/EBP-homologous protein; ROS: reactive oxygen species; DOX: doxorubicin; HSP: heat shock proteins; PKC-*α*: protein kinase C-alpha; *α*-FP: alpha-fetoprotein; CPK: checkpoint kinase 1; SOD: superoxide dismutase; HIF: hypoxia-inducible factor; GSH: glutathione; T-AOC: total antioxidant capability; AC-H: acetylation of histone H.

**Table 2 tab2:** Baicalein in HCC with possible mechanisms discussed in this paper.

Subfamily	Flavonoids	Typical origin	*In vitro*/*in vivo* Cell lines/modes Effective dose	Effects and related mechanisms	Author (year) References
Flavones	Baicalein	*Scutellaria baicalensis* (Scutellaria radix) roots (Sho-Saiko-To)	*In vitro* HepG2 cells 0, 12.5, 25 and 50 *μ*M	↑ protective autophagy, ↓ AKT/mTOR pathways ↓ p-AKT, p-ULK1, and p-4EBP1	Wang et al. (2015) [20]

			*In vitro* SMMC-7721 and Bel-7402 100 *μ*M	↑ apoptosis via ER stress, possibly by ↓ Bcl-2 family, ↑ intracellular calcium, and ↑ JNK ↑ protective autophagy	Wang et al. (2014) [93]

			*In vitro*/*in vivo* H22, Bel-7404, and Hep G2/cancer inducible ICR mice 0.5–100 *μ*g/mL/50 and 100 mg/kg	↑ G0/G1 cell cycle arrest, ↓ cancer cell proliferation, ↓ AKT ↓ *β*-catenin, and ↓ cyclin D1	Zheng et al. (2014) [21]

			*In vitro*/*in vivo* MHCC97H cells/nude mouse model LCI-D20 0, 10, 20, and 30 *μ*M/10 mg/kg/day	↓ tumor cell invasion, ↓ metastasis by ↓ cell motility ↓ migration via ↓ ERK pathway, ↓ MMP-2, ↓ MMP-9, ↓ u-PA expression, ↑ TIMP-1, and ↑ TIMP-2 expression	Chen et al. (2013) [37]

			*In vitro*/*in vivo* HepG2/HCC xenografts in mice 40–120 *μ*M/N/A	↑ apoptosis, ↓ MEK1, ↓ ERK1/2, ↓ Bad, ↓ MTP, ↑ caspase-9, and ↑ caspase-3	Liang et al. (2012) [76]

			*In vitro*/*in vivo* HA22T/VGH and SK-Hep1 cells/BALB c-nu mice 10 *μ*M/5, 10, and 20 mg/kg/day	↓ adhesion, invasion, migration, and proliferation ↓ metastasis; ↓ MMP-2, ↓ MMP-9, and ↓ uPA, ↓ p50 and p65 nuclear translocation, and ↓ pI*κ*B-*β* ↓ pPKC*α* and p38 MAPK	Chiu et al. (2011) [94]

			*In vitro* Hep J5 cells 10–100 *μ*M	↑ G2/M arrest and ↑ apoptosis (ER-dependent) ↑ cytochrome c release, ↑ caspase-9 and caspase-3, ↑ Bax/Bcl-2 ratio, ↑ AIF, and Endo G release from mitochondria	Kuo et al. (2009) [95]

			*In vitro* Hep G2 cells 25, 50, and 100 *μ*M	↑ G2/M population in Hep G2 cells ↑ apoptosis and ↓ proliferation	Chang et al. (2002) [96]

For abbreviations see footer of Table 1.

**Table 3 tab3:** Oroxylin A in HCC with possible mechanisms discussed in this paper.

Subfamily	Flavonoids	Typical origin	*In vitro*/*in vivo* Cell lines/modes Effective dose	Effects and related mechanisms	Author (year) References
Flavones	Oroxylin A	*Scutellaria baicalensis* Georgi	*In vitro* HepG2 25–100 *μ*M	↑ H_2_O_2_-triggered overactivation of the UPR pathway and ↓ AKT signaling	Xu et al. (2012) [23]

			*In vitro*/*in vivo* HepG2 cells/BALB/c nude mice 80 *μ*M/40 mg/kg; 80 mg/kg	↑ autophagy-mediated antitumor activity ↓ PI3K-PTEN-Akt-mTOR signaling pathway	Zou et al. (2012) [24]

			*In vitro* HepG2 cells 50, 100 and 200 *μ*M	↓ CAM-DR, ↓ integrin*β*1	Zhu et al. (2012) [80]

			*In vitro*/*in vivo* HepG2 cells/transplanted H22 mice 50 *μ*M/1,000 mg (kg/day)	↑ sensitivity of HepG2 cells to 5-FU by ↑ P53 and cleaved PARP; ↓ COX-2, ↓ Bcl-2, and ↓ pro-caspase-3	Zhao et al. (2010) [79]

			*In vitro* HepG2 cells 50, 100, and 200 *μ*M	↑ apoptosis by MAC-related mitochondrial pathway	Liu et al. (2009) [40]

			*In vitro* HepG2 50, 100, and 150 *μ*M	↑ apoptotic and G(2)/M phase arrest cells ↑ programmed cell death via ↓ Bcl-2 protein, ↓ pro-caspase-3 protein, and ↑ Bax protein	Hu et al. (2006) [97]

For abbreviations see footer of Table 1.

**Table 4 tab4:** Genistein in HCC with possible mechanisms discussed in this paper.

Subfamily	Flavonoids	Typical origin	*In vitro*/*in vivo* Cell lines/modes Effective dose	Effects and related mechanisms	Author (year) References
Isoflavones	Genistein	Dyer's broom (*Genista tinctoria*)	*In vitro* HepG2 cells 1.0 and 10 *μ*M	Regulation of MDR proteins, ↑ P-gp, and ↑ MRP2 protein; nutrient-drug interactions, ↑ chemoresistance to sorafenib	Rigalli et al. (2015) [31]

			*In vitro*/*in vivo* HepG2 cells/nude mice 3, 6, and 9 *μ*M/50 mg/kg BW	↑ E-cadherin, ↑ *α*-catenin, ↓ N-cadherin, and Vimentin ↓ intrahepatic metastasis by ↓ EMT, which was correlated with ↓ NFAT1	Dai et al. (2015) [22]

			*In vitro* HepG2, Huh-7, and HA22T3 dose dependent ≥20 *μ*M	↓ NF-*κ*B, ↓ AP-1 ↓ MAPK, I*κ*B, and ↓ PI3K/Akt, ↓ MMP-9, and ↓ EGFR	Wang et al. (2014) [98]

			*In vitro*/*in vivo* HCCLM3 cells/nude mice bearing human HCC xenografts 40 *μ*mol/L/2 mg/kg/day for 4 weeks	Synergistic effect with geneistin via ↓ cisplatin-induced MMP-2 expression	Chen et al. (2013) [37]

			*In vitro*/*in vivo* HepG2 and Hep3B/nude mice 15 M and 20 M/50 mg/kg	↑ ATO suppressive effect via ↓ NF-*κ*B pathway, ↓ cyclin D1, ↓ Bcl-xL, ↓ Bcl-2, ↓ c-myc, ↓ COX-2, and ↓ VEGF	Ma et al. (2011) [99]

			*In vitro* SK-Hep-1 cells 0–50 *μ*M	↑ apoptosis ↑ Fas, ↑ FasL, and ↑ p53; ↑ Bcl-2, ↑ caspase-9 and ↑ caspase-3, and ↑ PARP cleavage	Fang et al. (2010) [100]

			*In vitro*/*in vivo* HepG2, SK-Hep-1, and Hep3B/HepG2 xenografts in BALB/c nude mice 15 *μ*M, 15 *μ*M, and 20 *μ*M/50 mg/kg	Synergistic effect with ATO via ↓ Bcl-2 expression, ↑ Bax expression, ↑ caspase-9 and -3, ↑ cytochrome c, and ↑ ROS	Jiang et al. (2010) [101]

			*In vitro* Hep3B cells 12, 25, and 50 *μ*M	↓ p38MAPK and ↑ TRAIL-mediated apoptosis; ↓ ERK, ↓ MMP, ↑ caspase-3, caspase-8, and caspase-9 activity, ↑ PARP cleavage, and ↓ Bcl-2 protein	Jin et al. (2009) [39]

			*In vitro* Hep G2 cells 6.75 *μ*M baicalein + 25, 50, and 100 *μ*M silymarin	↑ cells in G0/G1 phase, ↓ S-phase ↑ Rb, ↑ p53, ↑ p21(Cip1) and ↑ p27(Kip1), ↓ cyclin D1, ↓ cyclin E, ↓ CDK4, and ↓ phospho-Rb	Chen et al. (2008) [102]

			*In vitro*/*in vivo* MHCC97-H cells/BALB/c nu/nu mice (LCI-D20) 5, 10, and 20 *μ*g/mL/50 mg/kg	↑ G(0)/G(1) cell cycle arrest, ↓ S phase, and ↑ apoptosis ↓ phosphorylation of FAK	Gu et al. (2009) [78]

			*In vitro* Hep3B cells 100 *μ*M	↑ ER stress and ↑ mitochondrial insult in Hep3B cells, ↑ m-calpain, ↑ GADD153, ↑ GRP78, and ↑ caspase-12 ↑ caspase-2, ↑ ROS, ↓ Mcl-1	Yeh et al. (2007) [77]

For abbreviations see footer of Table 1.

**Table 5 tab5:** Galangin in HCC with possible mechanisms discussed in this paper.

Subfamily	Flavonoids	Typical origin	*In vitro*/*in vivo* Cell lines/modes Effective dose	Effects and related mechanisms	Author (year) References
Flavonols	Galangin	*Alpinia officinarum *	*In vitro* HepG2 cell 74, and 148 *μ*M	↑ autophagy via ↑ TGF-*β* receptor/Smad pathway	Wang et al. (2014) [25]

			*In vitro* HepG2, Hep3B, and PLC/PRF/5 cells 134.0, 87.3, and 79.8 *μ*M for 24 h	↑ MAPKs involved in ER stress with ↑ GRP94, ↑ GRP78 and ↑ CHOP, and ↑ free cytosolic Ca^2+^ concentration	Su et al. (2013) [103]

			*In vitro* HepG2 cells Autophagy (130 *μ*mol/L) and apoptosis (370 *μ*mol/L)	↑ apoptosis, ↑ autophagy through a p53-dependent pathway	Wen et al. (2012) [81]

			*In vitro* HepG2, Hep3B, and PLC/PRF/5 46.25–370.0 *μ*mol/L	↑ apoptosis through a mitochondrial pathway ↑ AIF, ↑ cytochrome c	Zhang et al. (2010) [104]

For abbreviations see footer of Table 1.

**Table 6 tab6:** Quercetin in HCC with possible mechanisms discussed in this paper.

Subfamily	Flavonoids	Typical origin	*In vitro*/*in vivo* Cell lines/modes Effective dose	Effects and related mechanisms	Author (year) References
Flavonols	Quercetin	Fruits, vegetables, leaves, and grains	*In vitro* HepG2 cells 10, 20, and 40 *μ*M	Apoptotic cell death by regulating cell cycle and ↓ antiapoptotic proteins (Sp1 and Sp1 regulatory protein)	Lee et al. (2015) [27]

		Berries, grapes, potatoes, tomatoes, and onions Beverages (tea, wine, and bear)	*In vitro* HepG2 cell line 12.5–100 *μ*M	↑ G2/M phase arrest accompanied by an ↑ apoptotic cell death apoptosis via ↑ p53, ↑ p21, ↓ Cyclin D1, ↓ Cdk2, Cdk7 levels, and ↑ ROS	Li et al. (2014) [28]

			*In vitro* HepG2 cells 50 *μ*M	↑ p16-mediated cell cycle arrest and ↑ apoptosis	Zhao et al. (2014) [105]

			*In vivo* DEN induced liver cancer rats 10 mg/kg	↑ apoptosis, ↑ caspase 9 ↑ caspase 3, and ↑ PARP (the intrinsic mitochondrial pathway) ↓ lipid peroxidation ↓ NF*κ*B	Vásquez-Garzón et al. (2013) [106]

			*In vitro*/*in vivo* Hepatoma SMMC7721 cells and QGY7701 cells/athymic BALB/c nu/nu mice 0–150 *µ*M/100 mg/kg	↑ DOX-mediated apoptosis in p53-dependent hepatoma cells ↓ Bcl-xl	Wang et al. (2012) [83]

			*In vitro* HepG2 cells 50 *µ*mol/L	↓ HSPs in HepG2 cells	Zhou et al. (2011) [107]

			*In vitro* HepG2 50 *μ*M	↓ NF-*κ*B ↓ caspase-3, ↓ p38MAPK, unbalance of Bcl-2 proapoptotic and antiapoptotic proteins, ↑ JNK, and ↑ AP-1 regulation of survival/proliferation (AKT, ERK)	Granado-Serrano et al. (2010) [108]

			*In vitro* HA22T, VGH, and HepG2 cells 40 *μ*M	↑ oxidative stress, ↑ ROS ↑ apoptotic action of 2-methoxyestradiol	Chang et al. (2009) [109]

			*In vitro* HepG2 and SK-Hep1 20, 100, and 200 *μ*M	↑ TRAIL-induced apoptosis via ↑ Sp1-mediated DR5 and ↓ c-FLIPS	Kim et al. (2008) [110]

			*In vitro* HepG2 50 *μ*M	Tight regulation of survival/proliferation pathways via ↓ PI3/AKT, ↓ ERK, ↓ PKC-*α*, ↑ JNK, and ↑ PKC-delta	Granado-Serrano et al. (2008) [82]

			*In vitro* HepG2 cells 10–50 *μ*mol/L	↑ caspase-3, ↑ caspase -9, ↓ Bcl-xL/Bcl-xS, ↓ major survival signals, ↓ Akt, and ↓ ERK	Granado-Serrano et al. (2006) [42]

For abbreviations see footer of Table 1.

**Table 7:** Silibinin in HCC with possible mechanisms discussed in this paper.

Subfamily	Flavonoids	Typical origin	*In vitro*/*in vivo* Cell lines/modes Effective dose	Effects and related mechanisms	Author (year) References
Flavonolignans	Silibinin	Extract of the milk thistle seeds Milk thistle plant *(Silybum marianum*)	*In vitro* SNU761 cell line, Huh-BAT cell line 100 *μ*M	↓ EGFR-dependent Akt signaling ↑ growth-inhibiting effects of both gefitinib and sorafenib	Gu et al. (2015) [30]

			*In vitro*/*in vivo* HepG2 cells/male athymic nude mice 5, 10, or 20 *μ*M (↓ tumor cell adhesion, migration); 50, 100, and 200 *µ*M/200, 400 mg/kg/day (5 days/week)	↓ tumor cell adhesion, migration, ↓ GSH, ↓ T-AOC ↑ apoptotic index, ↑ caspase-3, and ↑ ROS ↓ notch signaling via ↓ Notch1 intracellular domain (NICD), ↓ RBP-J*κ*, and ↓ Hes1 proteins, ↑ Bax, ↓ Bcl2, ↓ survivin, and ↓ cyclin D1	Zhang et al. (2013) [29]

			*In vivo* HuH7 xenografts 80 and 160 mg/kg/day	↓ Ki-67 and ↓ *α*-FP production, ↓ NF-*κ*B content, ↓ polo-like kinase 1, ↓ Rb phosphorylation, and ↓ E2F1/DP1 complex ↑ p27/CDK4 complex and CPK 1 expression mediated by ↓ G1-S transition of the cell cycle ↓ survivin phosphorylation ↓ cell proliferation ↓ p-ERK, ↑ PTEN/PI(3)K/Akt, and ↑ ERK pathways ↑ AC-H3 and AC-H4 expression and ↑ SOD-1	Cui et al. (2009) [111]

			*In vitro* Hep3B 500 *μ*M	↓ cell proliferation ↓ HIF-1*α* and ↓ mTOR/p70S6K/4E-BP1 signalling pathway	García-Maceira et al. (2009) [112]

			*In vitro* HepG-2 25, 50 and 75 *μ*M	↓ cell proliferation, ↓ MMP 2 enzymatic activity, ↓ NO ↓ ERK 1/2, ↑ RKIP, Spred-1, and Spred-2 ↓ cell growth and proliferation via ↓ Hec1, and MMP-2	Momeny et al. (2008) [43]

			*In vitro* HuH7 cells 240 *μ*mol/L	↑ p21/CDK4 and p27/CDK4 complexes, ↓ Rb-phosphorylation ↓ E2F1/DP1 complex ↓ survivin ↑ caspase-3 and ↑ caspase-9 antiangiogenic effects via ↓ MMP2 and ↓ CD34 ↑ PTEN and ↓ p-Akt production ↑ AC-H3 and AC-H4	Lah et al. (2007) [113]

			*In vitro* HepG2 and Hep3B cells 100–300 *μ*mol/L	↑ G1 arrest in HepG2 and ↑ G1 and G2-M arrests in Hep3B cells ↑ Kip1/p27, ↓ cyclin D1, ↓ cyclin D3, ↓ cyclin E, ↓ CDK-2, and ↓ CDK4 ↓ G2-M regulators, ↓ CDK2, ↓ CDK4, and ↓ CDC2 kinase activity	Varghese et al. (2005) [114]

For abbreviations see footer of Table 1.

## Abbreviations

HCC: Hepatocellular carcinoma
EGCG: (−)-Epi-gallocatechin-3-gallate
EGC: (−)-Epi-gallocatechin
IGF: Insulin-like growth factor
IGF-1R: Type 1 insulin-like growth factor receptor
PI3K: Phosphatidylinositol 3-kinase
p-: Phosphorylated
STAT3: Signal transducer and activator of transcription 3
AMPK: AMP-activated protein kinase
TSC: Tuberous sclerosis complex
ERK: Extracellular signal regulated kinases
mTOR: Mammalian target of rapamycin
4E-BP1: Eukaryotic initiation factor 4E-binding protein-1
PARs: Proteinase-activated receptors
MAPK: Mitogen-activated protein kinase
ER: Endoplasmic reticulum
FADD: Fas-associated death domain
FLICE: FADD-like IL-1*β*-converting enzyme
c-FLIP: Cellular FLICE-inhibitory protein
Fas-L: Fas ligand
TNF: Tumor necrosis factor
TRAIL: TNF-related apoptosis-inducing ligand
EGFR: Epidermal growth factor receptor
NF-*κ*B: Nuclear factor kappa-light-chain-enhancer of activated B cells
UPR: Unfolded protein response
ATF6: Activating transcription factor 6
TGF-*β*: Transforming growth factor-*β*
EMT: Epithelial-to-mesenchymal transition
ECM: Extracellular matrix
PGE: Prostaglandin
HIF-1: Hypoxia-inducible factor 1
MMP: Matrix metalloproteinase
FGF: Fibroblast growth factor
AIF: Apoptosis-inducing factor
Endo G: Endonuclease G
JNK: c-Jun NH2-terminal kinase
GSK-3*β*: Glycogen synthase kinase-3*β*
ERK: Extracellular signal regulated kinases
IB: I-Kappa-B
u-PA: Urokinase-type plasminogen activator
TIMP: Tissue inhibitor of metalloproteinase
PARP: Poly ADP-ribose polymerase
ROS: Reactive oxygen species
ATO: Arsenic trioxide
NFAT1: Nuclear factor of activated T cells 1
MDM2: Mouse double minute 2 homolog
5-FU: 5-Fluorouracil
H22: Murine hepatoma 22
PTEN: Phosphatase and tensin homolog
CAM-DR: Cell adhesion mediated drug resistance
DAPK: Death-associated protein kinase
TPA: 12-*O*-Tetradecanoylphorbol-13-acetate
CDK: Cyclin-dependent kinases
DR5: Death receptor 5
HSPs: Heat shock proteins
VEGFR: Vascular endothelial growth factor receptor.
